# Cognitive rehabilitation for improving cognitive functions and reducing the severity of depressive symptoms in adult patients with Major Depressive Disorder: a systematic review and meta-analysis of randomized controlled clinical trials

**DOI:** 10.1186/s12888-023-04554-w

**Published:** 2023-01-27

**Authors:** Saba Mokhtari, Asieh Mokhtari, Farah Bakizadeh, Alireza Moradi, Mohammadreza Shalbafan

**Affiliations:** 1 Department of Psychiatry, University of Social Welfare and Rehabilitation Sciences, Tehran, Iran; 2 School of Allied Medical Sciences, Iran University of Medical Sciences, Tehran, Iran; 3 Department of Psychology, University of Tehran, Tehran, Iran; 4 Department of Clinical Psychology, Kharrazmi University, Tehran, Iran; 5 Institute for Cognitive Sciences Studies, Tehran, Iran; 6 Mental Health Research Center, Psychosocial Health Research Institute (PHRI), Department of Psychiatry, School of Medicine, Iran University of Medical Sciences, Tehran, Iran; 7 Brain and Cognition Clinic, Institute for Cognitive Sciences Studies, Tehran, Iran

**Keywords:** Cognitive Function, Major Depressive Disorder, Systematic Review, Meta-Analysis, Executive function, Verbal learning, Working memory

## Abstract

**Introduction:**

Nearly 40% of patients with Major Depressive Disorder (MDD) have been found to experience cognitive impairment in at least one domain. Cognitive impairment associated with MDD is disproportionately represented in patients that have not fully returned to psychosocial functioning. As awareness regarding cognitive dysfunction in MDD patients grows, so does the interest in developing newer treatments that specifically address these deficits.

**Method:**

In the present study, we conduct a systematic review of controlled randomized clinical trials that used cognitive training and remediation interventions for improving cognitive functions and reducing symptom severity in adult patients with MDD. We selected studies published before March 2022 using search databases including PubMed, ScienceDirect, Scopus, and Google scholar. For conducting the meta-analysis, standard differences in means with the random effect model and with a 95% confidence interval of change in outcome measures from baseline to post-intervention between the cognitive rehabilitation and the control groups were calculated.

**Results:**

The database search resulted in identifying 756 studies of interest, which ultimately 15 studies with 410 participants in the cognitive rehabilitation group and 339 participants in the control group were included. The meta-analysis of the data extracted from these studies, shows a moderate and significant effect on the executive function (d = 0.59 (95% CI, 0.25 to 0.93) *p*-value = 0.001, I^2^ = 15.2%), verbal learning (d = 0.45 (95% CI, 0.12 to 0.78) *p*-value = 0.007, I^2^ = 0.00%), and working memory (d = 0.41 (95% CI, 0.18 to 0.64) *p*-value < 0.001, I^2^ = 33%) of MDD patients. Although, there were no significant difference between intervention and control group in attention (d = 0.32 (95% CI, -0.01 to 0.66) *p*-value = 0.058, I^2^ = 0.00%) or depressive symptoms.

**Conclusion:**

This systematic review and meta-analysis indicate that cognitive rehabilitation is an effective intervention for the executive function, verbal learning, and working memory of MDD patients. Due to the importance of these neuropsychological deficits in day-to-day life and the core symptoms of MDD, cognitive rehabilitation should be considered an important part of treating MDD. Further research in this area and concentrated on these particular deficits is warranted.

## Introduction

MDD is considered a chronic, disabling, and (in most cases) recurring psychiatric condition [1] which is characterized by depressed mood, reduced interest and pleasure in daily activities, weight fluctuation, sleep and psychomotor distress, fatigue, feelings of worthlessness, trouble concentrating, and suicidal ideation [2, 3].

Depression is a major public health concern, with worldwide estimates indicating that 10.8% of individuals suffer from this chronic condition at some point in their lives [4]. Based upon estimations from the World Health Organization (WHO), MDD is responsible for the greatest share of burden linked to non-fatal health outcomes and accounts for nearly 12% of total years lived with disability [5].

Cognitive impairment is a common and frequent symptom of MDD. Nearly 40% of people who have currently or formerly been diagnosed with depression have been found to experience cognitive impairment in at least one domain. Cognitive impairment associated with MDD is disproportionately represented in patients that have not fully returned to psychosocial functioning [6, 7] and cannot be considered an epiphenomenon entirely secondary to signs of low mood [8].

Impaired cognitive function in patients with MDD does not prove to be limited to the acute phase of depression but persists when MDD has remitted [9]. Deficits in selective attention, working memory, long-term memory, verbal and visuospatial memory, attention, and processing speed, executive functioning, and verbal fluency remain persistent in remission from a depression episode and the level of cognitive impairment appears to worsen with repeated episodes [10–14]. These cognitive symptoms seem to have a significant impact on patients’ function and quality of life, interfere with their ability to contribute actively to the society, by sustaining employment or schooling, and risk the recurrence of their depression [15, 16].

Little is known about how such deficits arise in MDD. Current theories indicate that neuroanatomic changes in MDD patients’ brains might be the cause of observed deficits [17]. Neuroimaging studies show abnormal physical changes in the hippocampus, amygdala, caudate nucleus, putamen, and frontal cortex [18] and postmortem studies have shown a reduction in synaptic proteins in subgenual and/or glia and neural size, orbital and dorsolateral prefrontal cortex and amygdala [19–21].

Many studies indicate the effect of antidepressant drugs on cognitive impairment symptoms (in MDD patients) such as processing speed, working memory, visuospatial skills, sustained attention, etc., to be very small or statically non-significant [22] and overall, the data show that most of the standard treatments for MDD result in improved cognition. Although, the evidence continues to be limited by the small number of studies on this matter and small sample sizes and can’t be considered conclusive [23].

Current evidence indicates that cognitive interventions that are generally defined as cognitive remediation, training, and rehabilitation can show significant, albeit modest, improvements in cognitive functions such as attention, problem-solving, and memory across a range of mental illnesses and have been beneficial to patients suffering from anxiety disorders, Schizophrenia, ADHD, etc. [24–27].

Unfortunately, despite the growing recognition of the clinical importance of cognitive impairment in MDD, a major lack of consensus regarding clinical monitoring strategies persists as a barrier to clinicians. As awareness regarding cognitive dysfunction in MDD grows, so does the interest in developing newer treatments that specifically address these deficits [23].

Fortunately, present approach to treatment of psychiatric disorders is not limited to symptom management and has a major focus on functional abilities [28]. Hence, therapeutic interventions for improving cognitive function, including cognitive rehabilitation, have been growing and trending over past decade [29]. As a result, various models of cognitive rehabilitation have been developed and have been widely used for psychiatric disorders, such as MDD. The goal of these interventions was primarily focused on improving cognitive functions and then generalized on symptoms severity and daily functioning [30]. Since cognitive rehabilitation interventions are extensively growing and the research on the effectiveness of them on psychiatric disorders (other than schizophrenia) are relatively recent [30, 31], review study to verify this matter seems necessary.

In the present study, we aimed to conduct a systematic review of research projects that used cognitive training and remediation interventions for improving cognitive functions and reducing symptom severity in adult patients with MDD. This study is designed to determine the quality of evidence and the effectiveness of cognitive rehabilitation in the treatment of various cognitive impairments and also reducing the severity of MDD symptoms.

## Methods

### Eligibility

This article follows the guidelines of Preferred Reporting Items for Systematic Reviews and Meta-Analyses (PRISMA) [32].

Patients participating in studies involved in this systematic review and meta-analysis had at least 18 years of age and were clinically-defined current or lifetime history of MDD, using established criteria such as the Diagnostic and Statistical Manual of Mental Disorders 5^th^ edition guidelines (DSM-5). No geriatric research projects were included in the study.

Eligible studies included patients without any established neurodegenerative disease (e.g., dementia, Multiple system atrophy, etc.) or neurological condition (e.g., Parkinson’s disease, traumatic brain injury, etc.) in other words; participants whom their cognitive impairment is only derived from depression. All articles recruiting participants diagnosed with psychiatric illnesses (e.g., Schizophrenia) or specific neuropsychiatric conditions (e.g., stroke) were also excluded. Regarding studies with mixed diagnostic samples (e.g., patients with MDD and another group of patients diagnosed with Obsessive Compulsive Disorder), or mixed samples of preclinical depression and MDD articles, the research was included in our study if only the data of the patients with only MDD diagnosis could be extracted from the reports of the study.

The research projects which evaluate the effect of “cognitive remediation”, “cognitive rehabilitation”, and “cognitive training” were included and researches which assess effect of any other pharmacological or non-pharmacological treatment (e.g., Cognitive Behavioral Therapy or group therapies) were excluded.

Eligible studies included controlled randomized clinical trials involving at least two groups of eligible participants, with measures in pre- and post-intervention on at least one of the mood or cognition domains.

### Search strategy and study selection

**Fig. 1:**
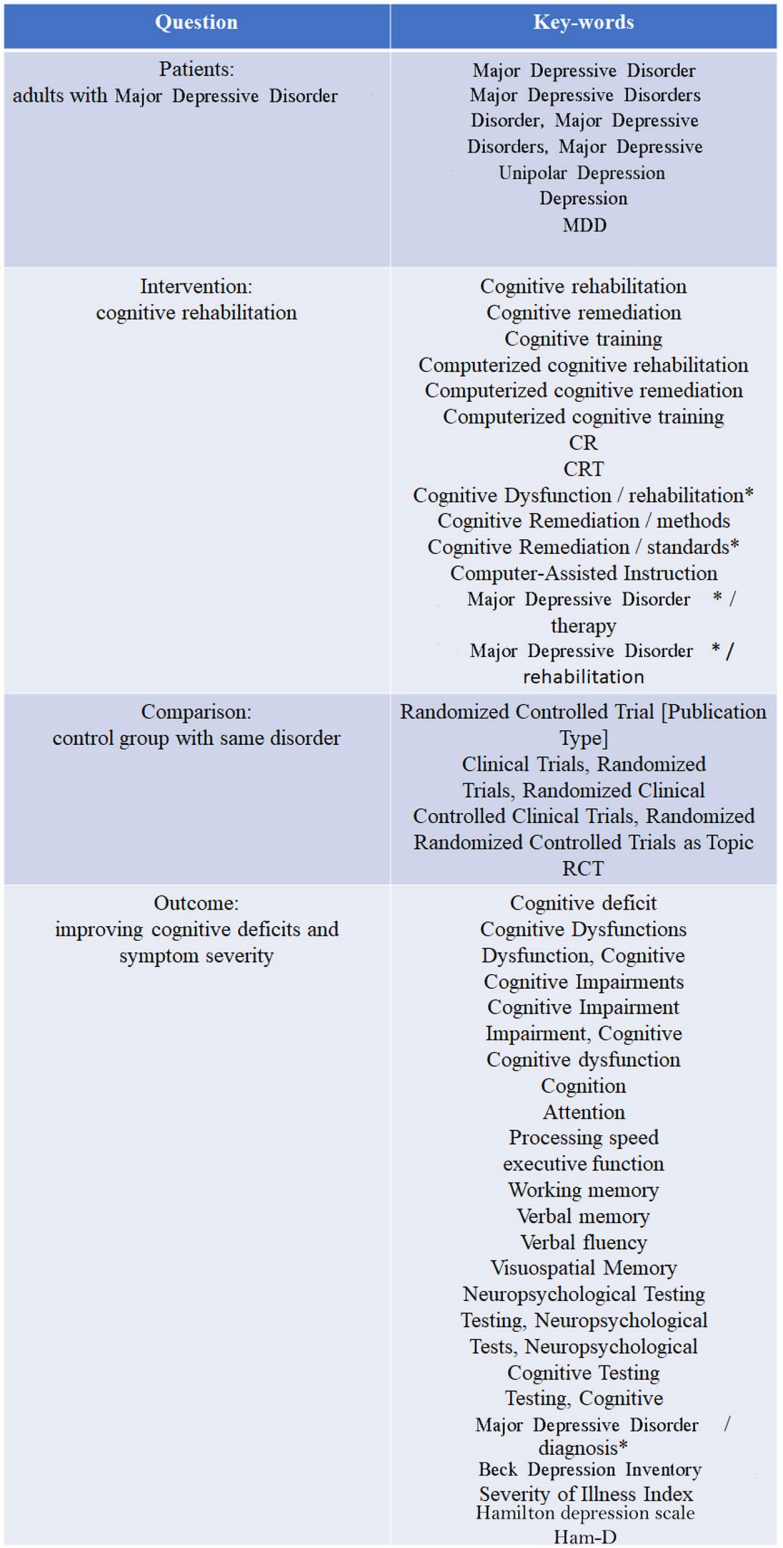
The details of the question and the key-words

Using search databases including PubMed, ScienceDirect, Scopus, and Google scholar alongside with collaborating with an expert research librarian we selected controlled and randomized (either blinded or not) trials published before march 2022. The literature search and study selection procedure and the terms used in our systematic search are illustrated in Fig. 1.

Our search and the primary review were conducted by our librarian. Then the first and second author reviewed abstracts and full texts. In the case of disagreement, a third researcher (the corresponding author) made the decision.

### Data analysis

For conducting the meta-analysis, we used Comprehensive Meta-Analysis Software (version 3) [33]. Standard differences in means with the random effect model and with a 95% confidence interval of change in outcome measures from baseline to post-intervention between the cognitive rehabilitation and the control groups were calculated.

Symptom severity of MDD and cognitive functions were included in the analysis as outcome measures. Standard tests for measuring MDD symptoms and objective standardized cognitive tests were eligible as the outcome. Cognitive functions were divided into standard cognitive domains and each domain used in more than two studies was included in the analysis.

Definition of weighted mean effect size were: 0.2–0.49 as small; 0.5–0.79 as medium; and > 0.8 as large [34]. The chi-square statistic and calculation of I^2^ were used to evaluate the heterogeneity across studies. I^2^ < 40% was defined as small, 30–60% moderate, 50–90% substantial and I^2^ > 75% as considerable heterogeneity [35]. Moreover, we applied sensitivity analysis and/or subgroup analysis in case of clinical heterogeneity.

Publication bias of studies was performed by inspecting funnel plots and we also evaluate the risk of bias in the studies for six main biases considering the Cochrane “Risk of bias” tool [36].

## Results

### Study characteristics

**Fig. 2:**
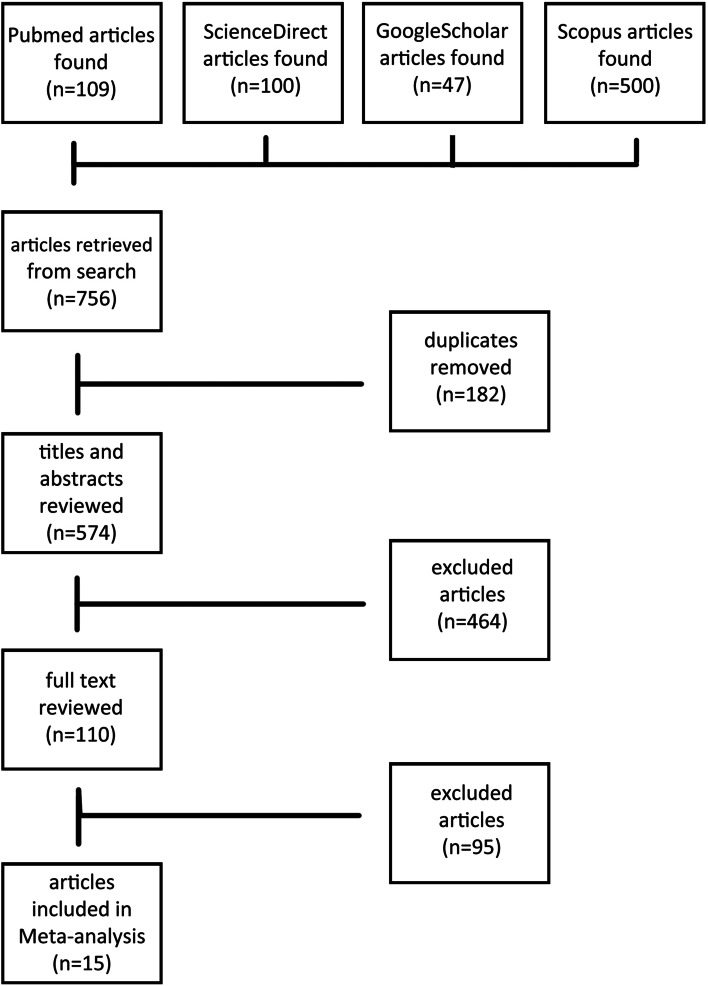
The flowchart of studies

The database search identified 756 studies of interest, initially of which 182 duplications were discarded from them. From 574 remaining records, 464 studies were removed by evaluating the titles and abstracts and from 110 full texts of articles, 15 studies met the inclusion criteria and were eligible for the study (Fig. 2). These studies contain 410 participants in the cognitive rehabilitation group and 339 participants in the control group. Nine studies used cognitive training interventions and six studies evaluated cognitive remediation. The reported data and type of evaluation in these 15 studies consisted of 13 studies that reported MDD symptoms’ severity, five studies reported attention, five studies reported executive function, five studies reported verbal learning, and nine studies reported working memory. The studies’ details are described in Table 1.

**Table 1:** Controlled clinical trials reporting cognitive rehabilitation in adults with MDD

Study	Participants	Intervention	Outcome
Areann 2016 [37]	132 adults with a PHQ-9 score > 10: 1- Experimental group (*n* = 56) 2- Control group (*n* = 76)	1- Experimental group: Project EVO (a cognitive training app) 2- Control group: an app that provided daily health tips	No post-intervention significant difference between groups: *P*-value = 0.15
Au 2021 [38]	22 patients with MDD: 1- Experimental group (*n* = 12) 2- Control group (*n* = 10)	1- Experimental group: occupational therapy and Chinese version of the PM module of the Cognitive Compensatory Training (CCT-C-PM) 2- Control group: occupational therapy	• ͰDepression anxiety stress scale score (DASS) Group*Time interaction F = 1.35, *P* = .99 • Cambridge prospective memory test score (CAMPROMPT-C) Group*Time interaction F = 7.97, *P* = .01
Bowie 2013 [39]	33 patients with treatment resistant MDD: 1- Experimental group (*n* = 17) 2- Control group (*n* = 16)	1- Experimental group: cognitive remediation (CR) 2- Control group: wait list Assessments: • Attention and processing speed: The symbol coding task, the continuous performance test, controlled oral word association test, Animal naming test, and Trail making test • Verbal Memory: The Hopkins verbal learning Test • Executive functioning: The letter number sequencing test, trail making test, and The Stroop color-word test	• Attention and information processing speed Group*Time interaction: F[1, 18] = 7.8, *p* = 0.012 • Verbal learning and memory Group*Time interaction: F[1, 16] = 6.2, *p* = 0.023 • Executive functioning Group*Time interaction: F[1, 17] = 0.8, *p* = 0.38
Ferrari 2021 [40]	115 MDD inpatients: 1- Experimental group (*n* = 56) 2- Control group (*n* = 59)	1- Experimental group: Cognitive Control Training (CCT) 2- Control group: comparable sham-training	• The Beck depression inventory (BDI-II) Group*Time interaction: F[1, 107] = 0.04, *p* = 0.84 • the random number generation task (RNGT) Group*Time interaction: F[3, 108] = 1.91, *p* = 0.13
Hoorelbeke 2016 [41]	68 patients with remitted MDD: 1- Experimental group (*n* = 34) 2- Control group (*n* = 34)	1- Experimental group: Cognitive Control Training (CCT) 2- Control group: a low cognitive load training	• The Beck depression inventory (BDI-II) Group*Time interaction: F[2, 65] = 1.2, *p* = 0.3 • Remission of depression questionnaire (RDQ) Group*Time interaction: F[1, 66] = 11.21, *p* = 0.001 • Non-adaptive PASAT accuracy Group*Time interaction: F[1, 66] = 18.52, *p* < 0.001
Klojčnik 2021 [42]	20 patients with MDD: 1- Experimental group (*n* = 10) 2- Control group (*n* = 10)	1- Experimental group: Standard rehabilitation interventions with computerized cognitive remediation (CCRT) 2- Control group: Standard rehabilitation interventions	• The Beck depression inventory (BDI-II) Group*Time interaction: F[1, 18] = 9.92, *p* = 0.006 • The test for attentional performance (TAP) Group*Time interaction: Alertness: F[1.18] = 10.57, *p* = 0.004 Inhibition: F[1.18] = 20.99, *p* < 0.001 Flexibility: F[1.18] = 9.18, *p* = 0.007 Divided attention: F[1.18] = 10.18, *p* = 0.005 • The tower of London (TOL) Group*Time interaction: Total move score: F[1.18] = 17.16, *p* = 0.001 Number of correct solutions: F[1.18] = 41.88, *p* < 0.001 Total problem-solving time: F[1.18] = 18.26, *p* < 0.001 Total execution time: F[1.18] = 6.38, *p* = 0.02 • The behavior rating inventory of executive function-adult (BRIEF-A) Group*Time interaction: Inhibition: F[1.18] = 1.1, *p* = 0.3 Shift: F[1.18] = 36.07, *p* < 0.001 Emotional control: F[1.18] = 20.21, *p* < 0.001 Initiate: F[1.18] = 23.56, *p* < 0.001 Working memory: F[1.18] = 6.07, *p* = 0.02 Plan/organize: F[1.18] = 5.39, *p* = 0.03
Lacoviello 2014 [43]	21 patients with unmedicated MDD: 1- Experimental group (*n* = 11) 2- Control group (*n* = 10)	1- Experimental group: The cognitive-emotional training group 2- Control group: Active control group	• The Hamilton depression rating scale (Ham-D) Group*Time interaction: F[1, 19] = 5.6, *P* = .02 • Unsignificant differences in: Digit-span forward (DSF), Digit-span backward (DSB), and Letter-number sequencing (LNS) subtests of the Wechsler adult intelligence scale
Listunova 2020 [44]	62 patients with partial remission MDD: 1- Experimental group (*n* = 38) 2- Control group (*n* = 19)	1- Experimental group: Cognitive remediation therapy (CRT) 2- Control group: Passive control group Assessments: • Attention: Alertness, divided attention and selective attention subscales of the test battery of the Vienna test • Learning and Memory: California verbal learning test (CVLT) and figural memory subscale of the test battery of the Vienna test • Executive functioning: Trail Making Test-L Version B, Tower of London-F, N-Back-verbal, and inhibition subscale of the test battery of the Vienna test	• Unsignificant differences in: The Hamilton depression rating scale (Ham-D), The Beck depression inventory (BDI-II), information processing speed, executive function, learning and memory • Attention Group*Time interaction: F[1, 35] = 5.232, *p* = 0.014
Moshier 2015 [45]	32 adults with a BDI-II score in 16 to 35 range: 1- Experimental group (*n* = 16) 2- Control group (*n* = 16)	1- Experimental group: Cognitive Control Training (CCT) 2- Control group: The peripheral vision task	• The Beck depression inventory (BDI-II) Group*Time interaction: F[1.6,60] = 0.64, *p* = 0.49 • The repeated knob-checking task Group*Time interaction: Memory accuracy: F[1, 64] = 0.45, *p* = 0.5 Memory confidence: F[1, 65] = 1.2, *p* = 0.27 Memory vividness: F[1, 65] = 2.66, *p* = 0.1 Memory detail: F[1, 65] = 2.67, *p* = 0.1
Moshier 2017 [46]	34 patients with MDD: 1- Experimental group (*n* = 17) 2- Control group (*n* = 17)	1- Experimental group: Computerized cognitive Control Training (CCT) 2- Control group: The peripheral vision task	• Unsignificant differences in the Beck depression inventory (BDI-II)
Semkovska 2015 [47]	24 MDD inpatients: 1- Experimental group (*n* = 12) 2- Control group (*n* = 12)	1- Experimental group: Computerized neurocognitive remediation therapy (NCRT) 2- Control group: Computer games	• Unsignificant differences in the Beck depression inventory (BDI-II)
Semkovska 2017 [48]	22 patients with remitted MDD: 1- Experimental group (*n* = 11) 2- Control group (*n* = 11)	1- Experimental group: Computerized neurocognitive remediation therapy (NCRT) 2- Control group: Computer games	• The Beck depression inventory (BDI-II) Group*Time interaction: F[1, 19] = 0.31 *P* = 0.59 • The Hamilton depression rating scale Group*Time interaction: F[1, 19] = 0.003 *P* = 0.96 • The Selective attention test Group*Time interaction: F[1, 19] = 7.05 *P* = 0.01 • The digit span backward test Group*Time interaction: F[1, 19] = 11.81 *P* = 0.003 • The logical memory test Group*Time interaction: Logical memory-I (total): F[1, 19] = 0.003 *P* = 0.96 Logical memory-II (retention): F[1, 19] = 14.36 *P* = 0.001 • The towers of London test Group*Time interaction: F[1, 19] = 26.9 *P* < 0.001 • The fluency switching test Group*Time interaction: F[1, 19] = 7.24 *P* = 0.01
Trapp 2016 [49]	41 MDD inpatients: 1- Experimental group (*n* = 21) 2- Control group (*n* = 20)	1- Experimental group: Cognitive remediation therapy (CRT) 2- Control group: Passive control group Assessments: • Depression: Beck depression inventory and Hamilton depression rating scale • Memory: visual reproduction II and logical memory II of Wechsler memory scale (WMS) • Working memory: WMS spatial span forward, WMS spatial span backward, WMS digit span forward, WMS digit span backward, WMS visual reproduction I, and WMS logical memory I • Attention: Degraded continuous performance test (CPT) omissions, Degraded CPT commissions, and Trail Making Test (TMT) A performance time • Executive function: WCST total errors, TMT B performance time, and TMT B-A	• Depression Group*Time interaction: F = 2.38 *P* = 0.13 • Attention Group*Time interaction: F = 0.62 *P* = 0.6 • Executive function Group*Time interaction: F = 3.14 *P* = 0.03 • Working memory Group*Time interaction: F = 2.63 *P* = 0.03 • Memory Group*Time interaction: F = 4.5 *P* = 0.03
Wanmaker 2014 [50]	61 adults with a BDI-II ≥ 10: 1- Experimental group (*n* = 34) 2- Control group (*n* = 27)	1- Experimental group: Working memory training 2- Control group: Video game	• The Beck depression inventory (BDI-II) Group*Time interaction: F[1, 46] = 0.63 *P* > 0.05 • The span board test Group*Time interaction: F[1, 46] = 8.98 *P* < 0.01
Wanmaker 2015 [51]	98 MDD patients (with or without anxiety) or GAD patients with at least moderate depressive symptoms (by BDI-II): 1- Experimental group (*n* = 49) 2- Control group (*n* = 49)	1- Experimental group: Working memory training 2- Control group: Video game	• The Beck depression inventory (BDI-II) Group*Time interaction: F[1,72] = 0.06 *P* > 0.05 • The digit forward test Group*Time interaction: F[1, 69] = 1.42 *P* > 0.05 • The digit backward test Group*Time interaction: F[1, 68] = 4.85 *P* < 0.05 • The reading span test test Group*Time interaction: F[1, 69] = 4.85 *P* < 0.05

### MDD symptoms’ severity

**Fig. 3:**
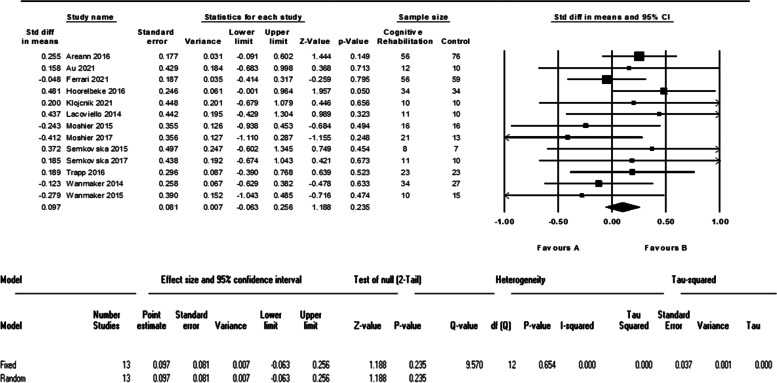
The forest plot of analyses of MDD symptoms’ severity

Thirteen studies, with 302 patients in the cognitive intervention group and 310 in the control group, evaluated the severity of MDD symptoms and reported the needed data. The meta-analysis of these data did not show a significant difference between intervention and control group (d = 0.09 (95% CI, -0.06 to 0.25) *p*-value = 0.23, I^2^ = 0.00%). The forest plot of analyses of MDD symptoms’ severity is shown in Fig. 3.

### Cognitive functions

**Fig. 4:**
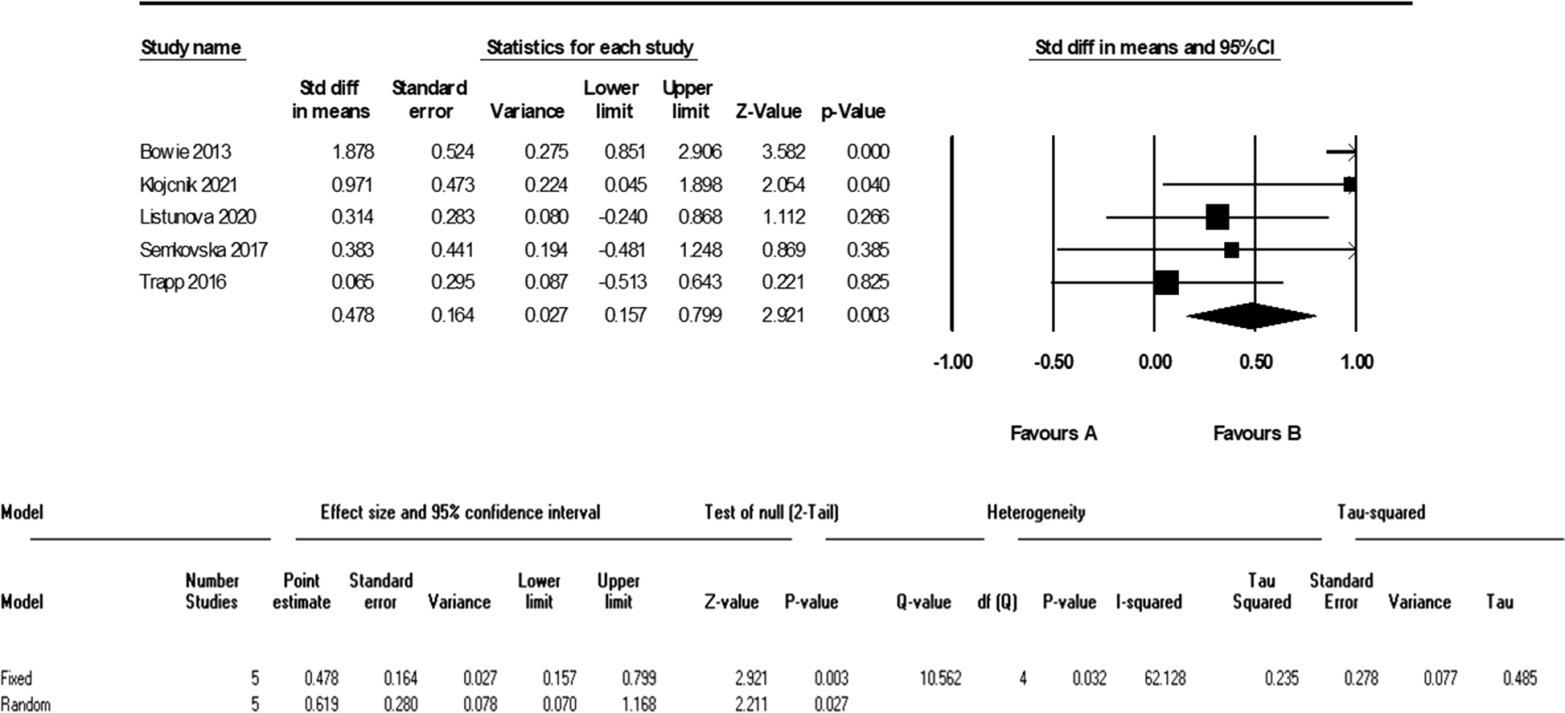
The forest plot of analyses of the attention

**Fig. 5:**
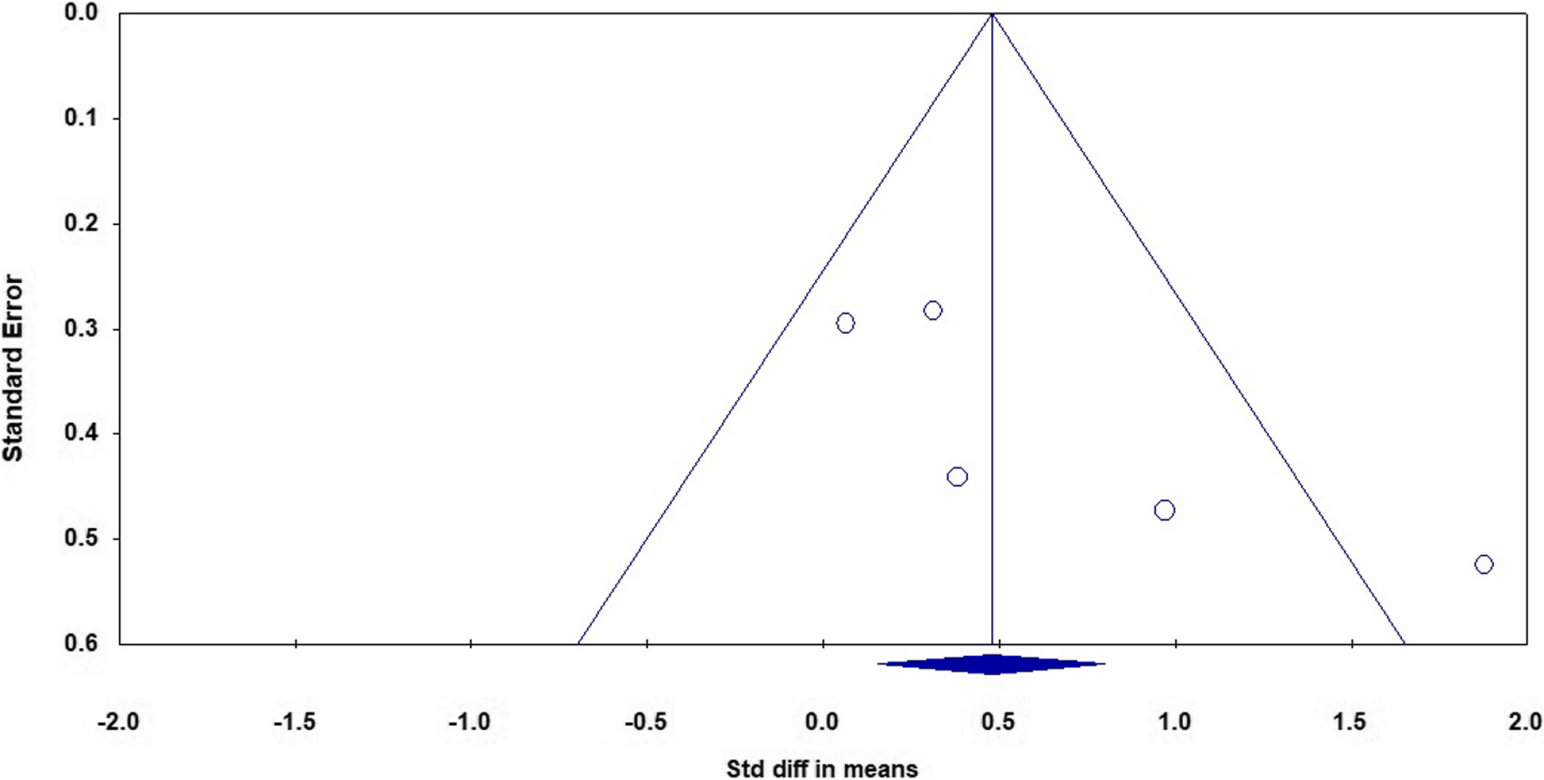
The funnel plot of analyses of the attention

After examining all included studies, we included attention, executive function, verbal learning, and working memory in the analyses. The meta-analysis of five studies that evaluated the attention of 93 patients in the cognitive intervention group and 72 patients in the control group showed an I^2^ = 62% and therefore we used the random effect model. The analysis showed a significant difference between the two groups with a moderate effect size (d = 0.61 (95% CI 0.07 to 1.16) *p*-value = 0.02). The forest plot of analyses of the attention is shown in Fig. 4 and the funnel plot of the analyses is shown in Fig. 5.

**Fig. 6:**
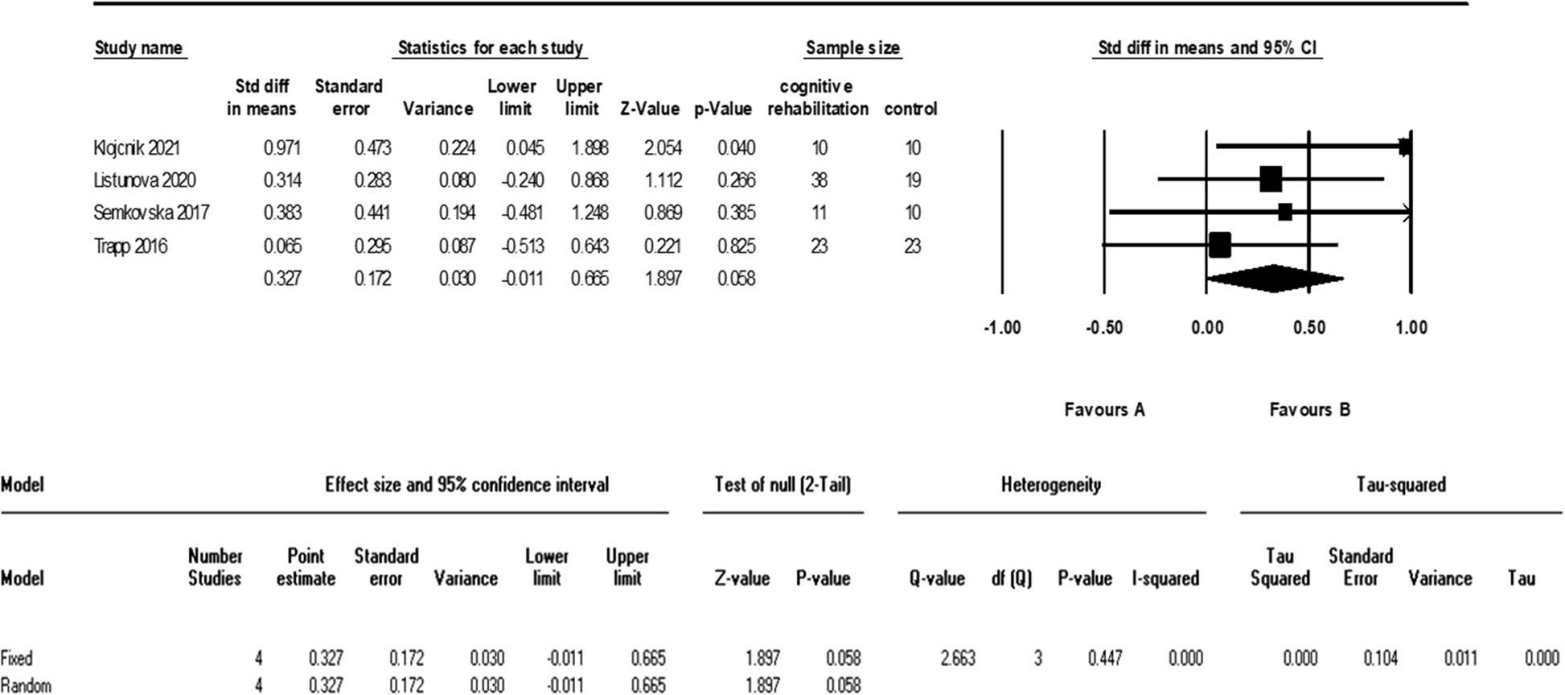
The forest plot of analyses of the attention after sensitivity analysis

As it is evident in the plots, sensitivity analysis (remove-one analysis) showed that Bowie et al. study has the outlier data. The meta-analysis without this study did not show a significant difference between intervention and control group (d = 0.32 (95% CI, -0.01 to 0.66) *p*-value = 0.058, I^2^ = 0.00%). The forest plot of this analysis is shown in Fig. 6.

**Fig. 7:**
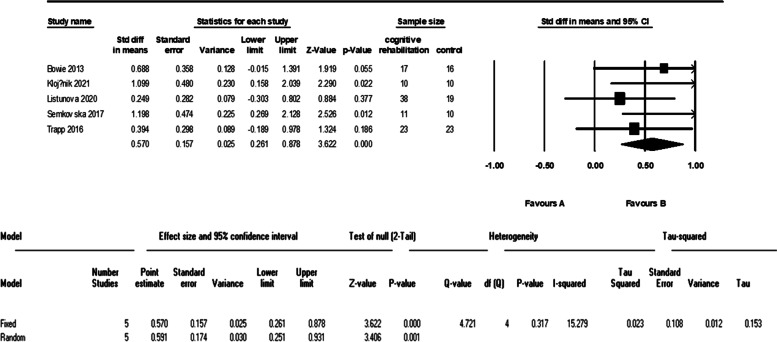
The forest plot of analyses of the executive function

Five studies evaluated the executive function of 99 patients in the cognitive intervention group and 78 patients in the control group. The meta-analysis of these data showed a significant difference between the two groups with a moderate effect (d = 0.59 (95% CI, 0.25 to 0.93) *p*-value = 0.001, I^2^ = 15.2%). The forest plot of analyses of the executive function is shown in Fig. 7.

**Fig. 8:**
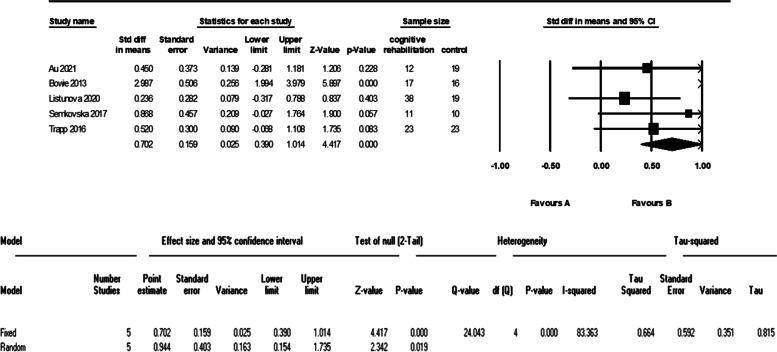
The forest plot of analyses of the verbal learning

**Fig. 9:**
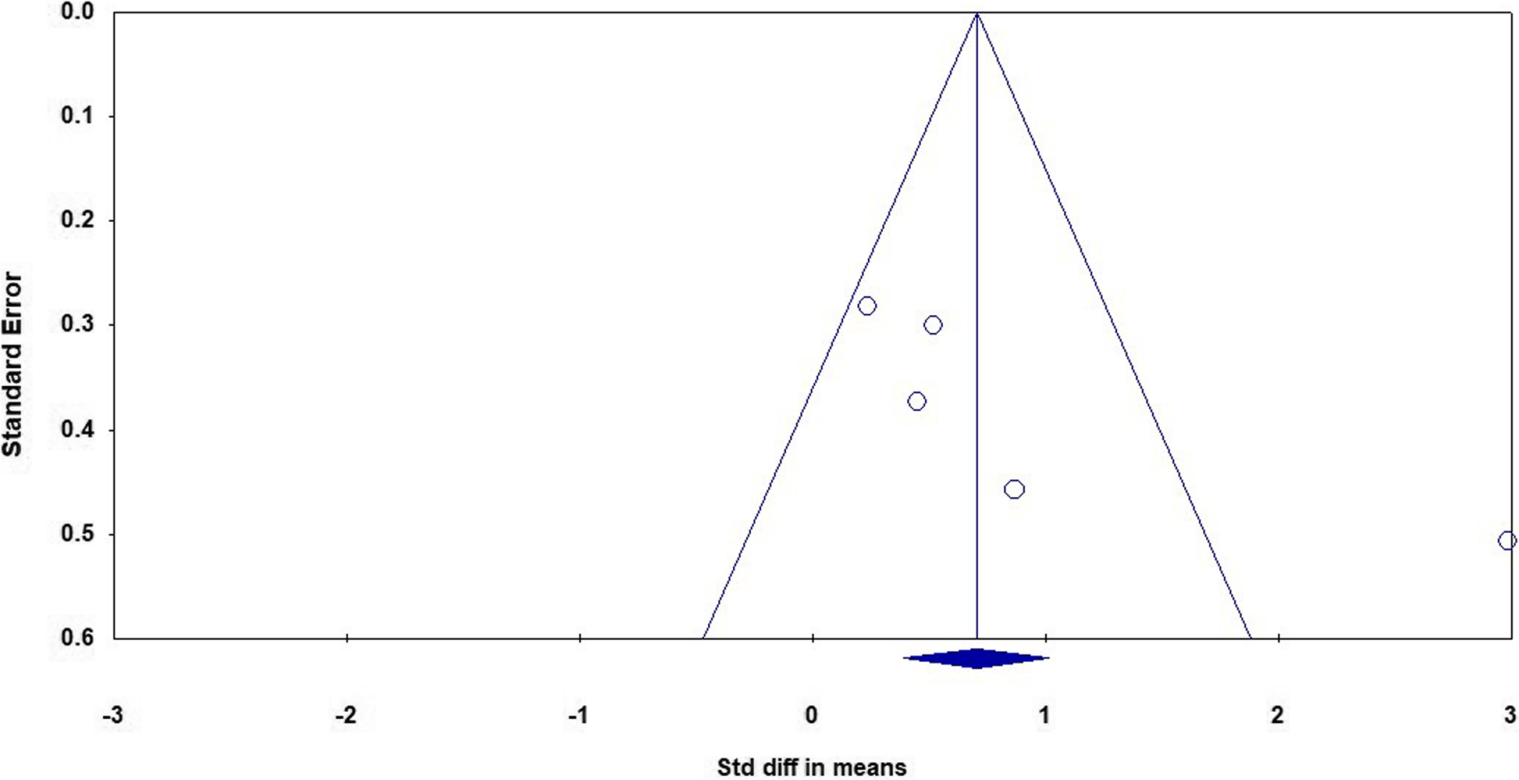
The funnel plot of analyses of the verbal learning

Five studies evaluated the verbal learning of 101 patients in the cognitive intervention group and 87 patients in the control group. The meta-analysis of these data showed an I^2^ = 83% and therefore we used the random effect model. The analysis showed a significant difference between the two groups with a large effect size (d = 0.94 (95% CI 0.15 to 1.73) *p*-value = 0.01). The forest plot of analyses of the verbal learning is shown in Fig. 8 and the funnel plot of the analyses is shown in Fig. 9.

**Fig. 10:**
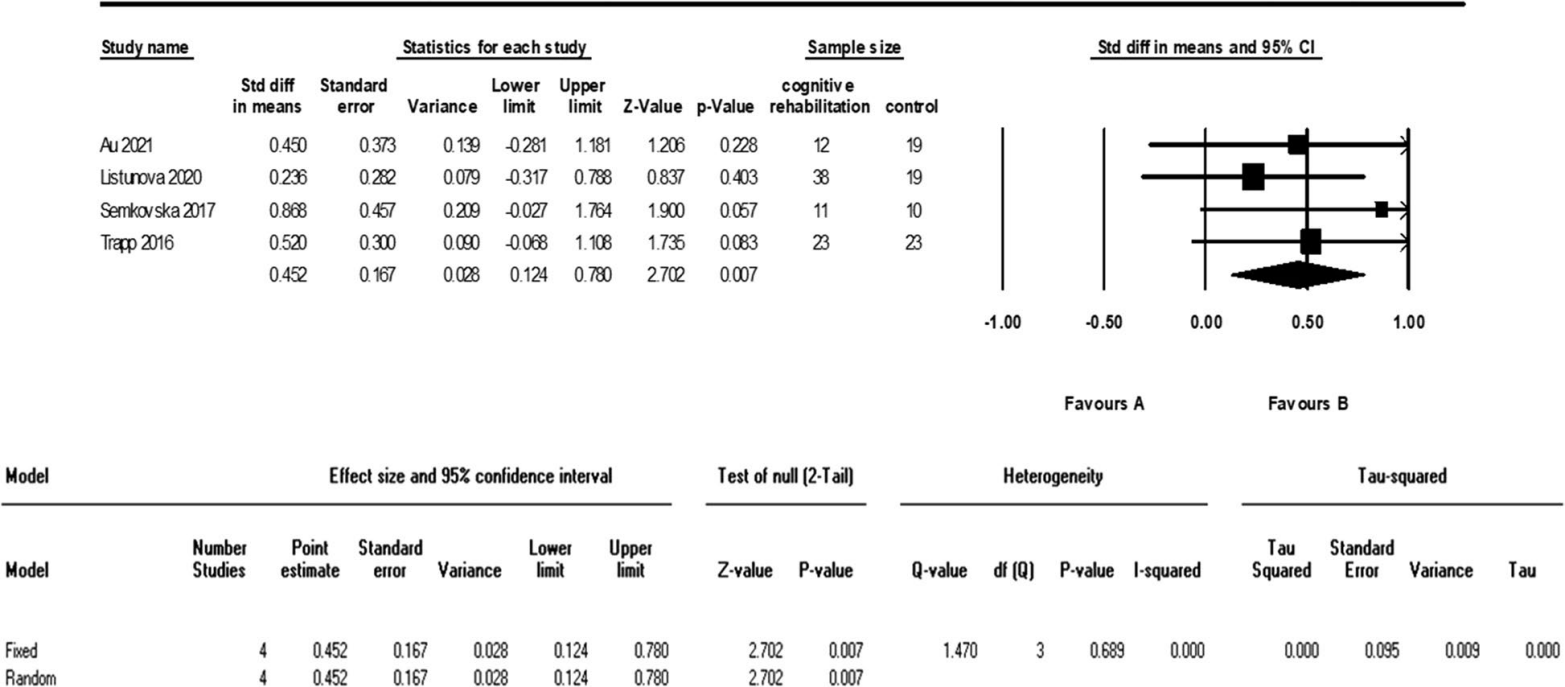
The forest plot of analyses of the verbal learning after sensitivity analysis

As it is shown in the plots and sensitivity analysis (remove-one analysis) Bowie et al. study has the outlier data. The meta-analysis without this study showed a significant difference between intervention and control group (d = 0.45 (95% CI, 0.12 to 0.78) *p*-value = 0.007, I^2^ = 0.00%). The forest plot of this analysis is shown in Fig. 10.

**Fig. 11:**
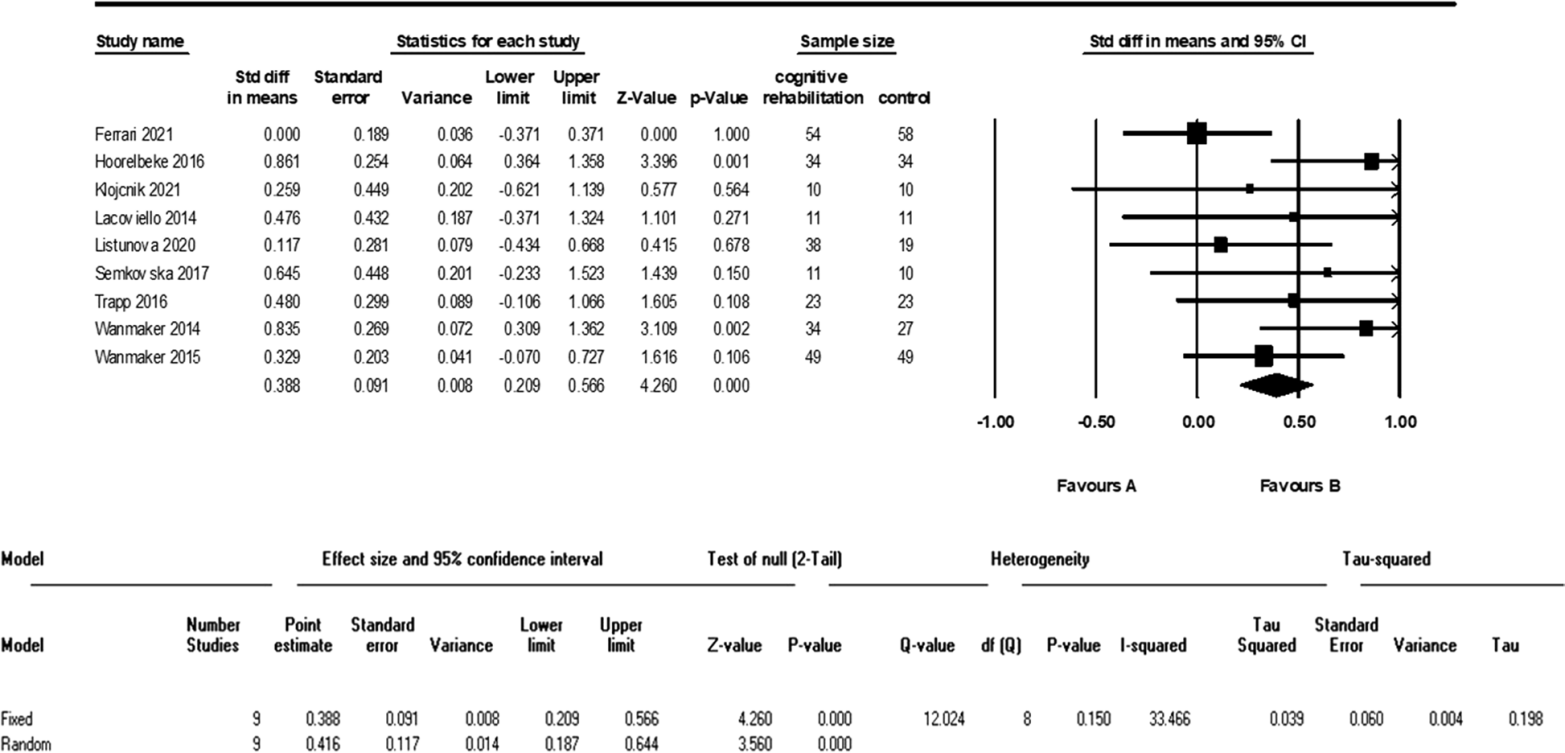
The forest plot of analyses of the working memory

Nine studies evaluated the working memory of 264 patients in the cognitive intervention group and 241 patients in the control group. The meta-analysis of these data showed a significant difference between the two groups (d = 0.41 (95% CI, 0.18 to 0.64) *p*-value < 0.001, I^2^ = 33%). The forest plot of analyses of the working memory is shown in Fig. 11.

### Subgroup analysis

We performed a sub-group analysis on the severity of MDD symptoms to determine the effect of the model of intervention (cognitive training and cognitive remediation).

The sub-group analysis of severity of MDD symptoms did not show a significant difference between the cognitive intervention and the control group in four studies that evaluated the cognitive remediation (d = 0.21 (95% CI, -0.16 to 0.6) *p*-value = 0.26, I^2^ = 0.00%), and 11 studies that evaluated the cognitive training (d = 0.06 (95% CI, -0.12 to 0.25) *p*-value = 0.5, I^2^ = 11%).

### Risk of bias

**Fig. 12:**
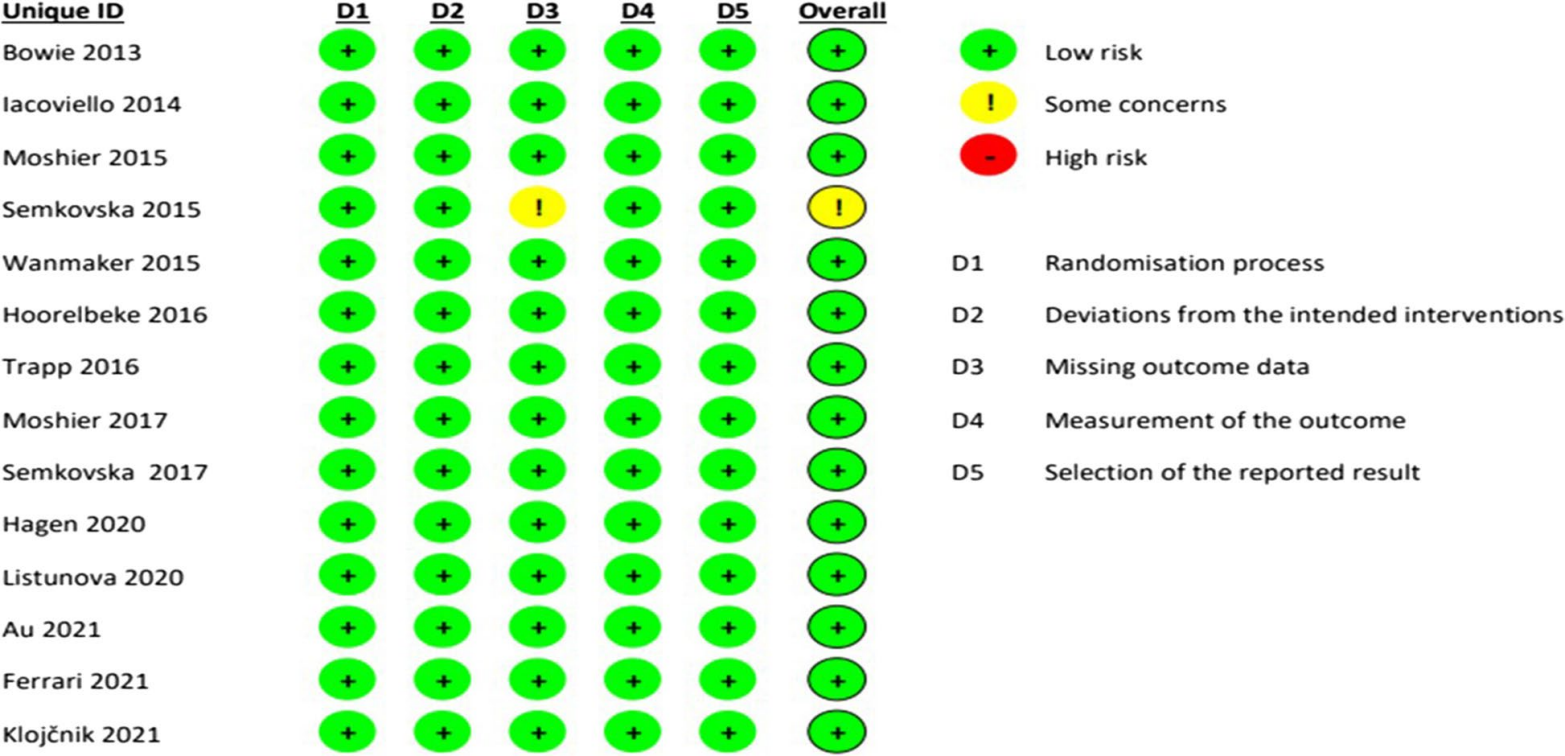
The assessment of the main biases of the studies

The evaluation of the risk of bias in the studies for six main biases showed that the quality of all the studies was relatively high. The result of the assessment of the main biases of the studies is shown in Fig. 12.

## Discussion

We conducted this systematic review and meta-analysis to evaluate whether cognitive rehabilitation is effective in terms of symptom severity and cognition in adult patients with MDD.

In summary, our study indicates that cognitive rehabilitation is an effective intervention for the executive function, verbal learning, and working memory of MDD patients. However, current cognitive rehabilitation therapies are not effective for improving attention, and in reducing the severity of symptoms of MDD patients.

A systematic search strategy revealed a noticeably larger number of studies on this matter, although 15 articles were standard randomized clinical trials with a control group and on adult patients with clinical criteria of MDD. The exclusion of a large number of studies with subclinical depressed patients has shown that cognitive rehabilitation is not at the center of attention for treating clinically diagnosed MDD, even though cognitive impairment associated with MDD is disproportionately represented in patients that have not fully returned to psychosocial functioning [6, 7]and cannot be considered an epiphenomenon entirely secondary to signs of low mood [8].

Based on the findings of this review, current cognitive rehabilitation therapies are not effective in reducing the severity of symptoms of MDD patients. This finding is not different between cognitive training and cognitive remediation therapies. Our finding is not consistent with the latest meta-analysis conducted on cognitive remediation and cognitive training in MDD patients. Woolf et al. [52] performed a meta-analysis of cognitive training in adults with MDD and reported a moderate and statistically significant effect on the severity of depressive symptoms. This difference can be due to including non-randomized control trials, a smaller number of studies (up to 2016) and larger heterogeneity (I^2^ = 40.6%) in Woolf et al. study. Legemaat et al. [9] conducted a meta-analysis on cognitive remediation in MDD patients and reported a small and statistically significant effect on the severity of depressive symptoms. The inconsistency of our findings can be due to including geriatric patients, non-randomized clinical trials, and larger heterogeneity (I^2^ = 40%) in the study of Legamaat et al.

The findings of our study did not show a significant effect of cognitive rehabilitation on the attention of adults with MDD. We performed the random effect model analysis because of I^2^ = 62% and reached a significant difference between the two groups with a moderate effect size. Despite that, due to the asymmetry of one study in the funnel plot and the results of sensitivity analysis (remove-one analysis), we excluded one study with outlier data. The analyses without this study showed no significant differences. Our findings are not consistent with recent meta-analysis on this matter even though, no systematic review has been conducted on both cognitive training and cognitive remediation in adults with MDD. The study of Legamaat et al. in 2021 showed a small and significant effect of cognitive remediation in MDD patients. This inconsistency can be due to aforementioned differences between our study and Legammat’s.

We found a moderate and significant effect on the executive function, verbal learning, and working memory of patients with MDD. All of the studies that evaluated executive function and were included in our analysis have used cognitive remediation techniques. Despite this fact, our findings were consistent with Woolf et al. (that evaluated cognitive training), and not similar to Legammat et al. (that evaluated cognitive remediation) which can be because of the differences mentioned before. The studies included in our analysis of verbal learning and working memory were from both cognitive remediation and cognitive training methods. Our findings in this matter are with less heterogeneity but are consistent with the study of Legammat et al. (I^2^ = 64%).

The importance and magnitude of deficit in executive function among MDD patients has been shown in different studies. Multiple studies have shown that executive dysfunction is strongly associated with some well-known aspects of MDD characteristics such as deficits in emotion regulation [53], attentional bias for negative stimuli [54], and rumination [55, 56] and can even give rise to other cognitive dysfunctions such as attention and problem-solving impairments [57]. Furthermore, executive function impairment remains among individuals with MDD even in remission and in euthymic phase [8, 58, 59]. The cognitive deficits in executive function and memory domains have been shown to be associated with occupational, social and global functioning and quality of life of patients with MDD [60]. Moreover, executive function, verbal learning and memory have been shown to be treatment outcome predictors in MDD [61] and a recent study indicated that executive dysfunction can be risk factor for suicide attempt and suicide preventive interventions should concentrate on executive function rehabilitation [62]. Considering all these significant aspects of verbal learning, memory and particularly executive function in adults with MDD and the significant effect of cognitive rehabilitation on these domains, clinical focus on cognitive rehabilitation is warranted.

Even though included studies used different methods of cognitive remediation and cognitive training for MDD patients, some similar and related methods are worth mentioning:

Five of the included studies (more than half of the studies that used cognitive training interventions) evaluated the Cognitive Control Training’s (CCT) effect on cognitive functions and symptom severity of patients with MDD. It has been shown that cognitive control impairment and emotion regulation deficits have a role in depression vulnerability and it has been suggested that improving cognitive control can be beneficial for treatment outcome of MDD patients [63, 64]. CCT is a modified trainings focuses on working memory to improve cognitive control [65]. CCT contains different tasks in this matter which the most frequently used tasks are the adaptive Paced Auditory Serial Addition Task (aPASAT), and the sustained attention training [65]. The suggested mechanism of effect of these tasks is an increase in activity of the dorsolateral prefrontal cortex (DLPFC), which is known to be under-activated in depression [66, 67]. Three of the included studies that used CCT, only evaluated the symptoms severity of patients and two of them assessed both symptom severity and the working memory of participants. Based on the findings of our study, similar to other cognitive rehabilitation interventions, CCT has a beneficial effect on working memory of MDD patients but it is not effective for MDD’s symptoms severity (d = 0.02 (95% CI, -0.28 to 0.33) *p*-value = 0.87, I^2^ = 30%)).

All of the included studies that evaluated cognitive remediation therapies, used computer-based models. Two of these studies used a training program named CogniPlus® which focuses on divided and selective attention, working memory, planning, response inhibition and alertness with a personalized task difficulty. Another two researches used a computerized intervention package named RehaCom as a neurocognitive remediation therapy. This software has been assessed and used on patients with schizophrenia [68] and neurological problems [69]. In the depression model, RehaCom focuses on divided attention, figural memory, verbal memory, and planning (plan a day and shopping [47, 48]). The suggested effect mechanism of both these computer-based models is mobilizing brain neuroplasticity and improving the synaptic communication between neurons by simulating the environmental change and learning new skills by repetitive practices [42, 47, 48, 70]. This mechanism can be a possible rational for improvement of the cognitive functions of MDD patients but it was not effective on their symptom severity.

The evaluation of included studies showed relatively high quality. As we included only randomized and controlled trials, there are no biases in these areas and the majority of studies were conducted concealed and blinded. Considering the moderate effect size and low risk of bias of almost all of the studies, these results can be reliable and conclusive.

Even though the number of included studies and their quality were satisfactory, there are some limitations in this study that is notable to mention. Only three studies evaluated a follow-up measure. However, the duration of follow-up was not similar (one month, three month and one year) and hence not suitable for analysis. This matter is important for many reasons. Besides the obvious value of evaluating the persistency of the effect of cognitive rehabilitation through time, the follow-up measure can be helpful to distinguish different factors of the treatment effect. For example, the mood improvement that is reported by many of the studies can be due to the feel of self-efficacy and self-confidence generated by involving in cognitive rehabilitation interventions and passing the structures and levels of cognitive tasks and consequently it will not show in follow-up measurements.

Another important matter and limitation of our study is that we couldn’t analyze the effect of MDD severity and many of included studies did not provide the history of MDD treatments and duration of participants’ diagnosis and our analysis is not homogenous on this matter. It can be an important factor since the effect of interventions can be inconsistent in different levels of severity and state of treatment. For example, it is possible that severely depressed patients or individuals in first stages of antidepressant treatment cannot fully engage in cognitive demanding tasks or the helplessness of chronic and treatment resistant patients can affect the results.

Mentioned factors should be considered in future studies. Additionally, further research should focus on executive function, verbal learning and working memory to provide further evidence and be of service to clinical practice.

## Conclusion

This systematic review and meta-analysis indicate that cognitive rehabilitation is an effective intervention for the executive function, verbal learning, and working memory of MDD patients. Due to the importance of these neuropsychological deficits in day-to-day life and the core symptoms of MDD, cognitive rehabilitation should be considered an important part of treating MDD. Further research in this area and concentrated on these particular deficits is warranted.

## Data Availability

The datasets used and analyzed during the current study are available from the corresponding author on reasonable request.
